# Gonadotropin-Releasing Hormone Stimulate Aldosterone Production in a Subset of Aldosterone-Producing Adenoma

**DOI:** 10.1097/MD.0000000000003659

**Published:** 2016-05-20

**Authors:** Rui Kishimoto, Kenji Oki, Masayasu Yoneda, Celso E. Gomez-Sanchez, Haruya Ohno, Kazuhiro Kobuke, Kiyotaka Itcho, Nobuoki Kohno

**Affiliations:** From the Department of Molecular and Internal Medicine, Graduate School of Biomedical and Health Sciences, Hiroshima University, Hiroshima, Japan (RK, KO, MY, HO, KK, KI, NK); and Division of Endocrinology, G.V. (Sonny) Montgomery VA Medical Center, University of Mississippi Medical Center, Jackson, MS, USA (CEG-S).

## Abstract

We aimed to detect novel genes associated with G protein-coupled receptors (GPCRs) in aldosterone-producing adenoma (APA) and elucidate the mechanisms underlying aldosterone production.

Microarray analysis targeting GPCR-associated genes was conducted using APA without known mutations (APA-WT) samples (n = 3) and APA with the KCNJ5 mutation (APA-KCNJ5; n = 3). Since gonadotropin-releasing hormone receptor (*GNRHR*) was the highest expression in APA-WT by microarray analysis, we investigated the effect of gonadotropin-releasing hormone (GnRH) stimulation on aldosterone production.

The quantitative polymerase chain reaction assay results revealed higher GNRHR expression levels in APA-WT samples those in APA-KCNJ5 samples (*P* < 0.05). LHCGR levels were also significantly elevated in APA-WT samples, and there was a significant and positive correlation between GNRHR and LHCGR expression in all APA samples (*r* = 0.476, *P* < 0.05). Patients with APA-WT (n = 9), which showed higher GNRHR and LHCGR levels, had significantly higher GnRH-stimulated aldosterone response than those with APA-KCNJ5 (n = 13) (*P* < 0.05). Multiple regression analysis revealed that the presence of the KCNJ5 mutation was linked to GNRHR mRNA expression (β = 0.94 and *P* < 0.01). HAC15 cells with *KCNJ5* gene carrying T158A mutation exhibited a significantly lower GNRHR expression than that in control cells (*P* < 0.05).

We clarified increased expression of GNRHR and LHCGR in APA-WT, and the molecular analysis including the receptor expression associated with clinical findings of GnRH stimulation.

## Abstract 2

Supplemental Digital Content is available in the text

## INTRODUCTION

Primary aldosteronism (PA) which is the most common cause of secondary hypertension is caused by an excessive and autonomous secretion of aldosterone, and is associated with cardiovascular morbidity and mortality.^1–4^ The 2 most common forms of PA are aldosterone-producing adenoma (APA) and idiopathic hyperaldosteronism, which is also known as bilateral adrenal zona glomerulosa hyperplasia.^1^ Excessive expression of steroidogenic enzymes, in particular aldosterone synthase which catalyzes the final steps of aldosterone biosynthesis, are necessary to produce aldosterone in APA.^5,6^ Activation of intracellular calcium signaling is crucially important for the molecular mechanisms of *CYP11B2*, the gene encoding aldosterone synthase, induction in APA.^5,7^

Although the pathogenesis of APA is unclear, somatic mutations of ion channels have been proposed to induce increases in aldosterone production and adenoma formation. Recent exome sequencing and clinical studies revealed that somatic mutations in the *KCNJ5* gene, which codes for the inwardly rectifying potassium channel Kir3.4, were found in 30% to 60% of APA cases.^8–11^ We and others have demonstrated that these mutations could be associated with autonomous aldosterone production via activation of intracellular calcium signaling and increases in *CYP11B2* expression levels.^8,12,13^ Following on from these findings, somatic *ATP1A1* mutations in 5.2% and *ATP2B3* mutations in 1.6% of APA cases were also detected. The genes *ATP1A1* and *ATP2B3* encode the Na/K-ATPase alpha subunit and Ca-ATPase, respectively.^14^ More recently, somatic mutations in *CACNA1D*, which encodes a voltage-dependent calcium channel, were identified in 5% to 9.3% of APA cases.^11,15,16^ According to in vitro studies, these mutations lead to an increase in intracellular calcium levels.^14–16^ Aldosterone production in 50% to 70% of APA cases are likely associated with these mutations; however, the etiology and pathogenesis of APA lacking mutations (APA without known mutations [APA-WT]) requires clarification.

Autonomous aldosterone production in some APAs could be the result of ectopic or aberrant G protein-coupled receptors (GPCRs).^17–20^ Ye et al^19^ reported that APA samples had higher mRNA expression levels of gonadotropin-releasing hormone receptor (GNRHR), luteinizing hormone/choriogonadotropin receptor (LHCGR), serotonin receptor 4, glutamate receptor metabotropic 3, endothelin receptor type B-like protein, and cosyntropin receptor compared with that in normal samples. Another research group demonstrated that some adenomas expressed higher levels of GNRHR and thyrotropin receptor mRNAs than those in normal tissue.^17^ Remarkably, some APAs exhibit increased expression levels of ectopic or aberrant GPCRs. Regulatory factors of GPCR expression in APA have not been elucidated as yet. In general, individual cells from different organs or cancers exhibit specific GPCR expression profiles, with GPCRs contributing to physiological regulation.^21^ Ectopic or aberrant GPCR expression could be related to the presence of previously described mutations, although there are no published studies to confirm it.

In this study, we proposed to identify the expression of specific GPCR or GPCR-related genes that might regulate aldosterone production in APA-WT using microarrays. We detected higher expression levels of GNRHR mRNAs in APA-WT sample compare with those in APA samples containing the KCNJ5 mutation (APA with the KCNJ5 mutation [APA-KCNJ5]). Consequently, we hypothesized that aberrant GNRHR and LHCGR expression might be associated with aldosterone production in APA-WT. We also demonstrated that aldosterone levels were increased as a result of stimulation with gonadotropin-releasing hormone (GnRH). Patients with APA-WT, which showed higher GNRHR and LHCGR levels, had strong GnRH-stimulated aldosterone response.

## METHODS

### Patients and Tissue Collection

The diagnosis of PA, and subtype diagnosis, was performed according to the guidelines from the Japan Endocrine Society (Supplementary data).^22^ First, 6 APA samples (wild type n = 3, KCNJ5 mutation n = 3) were enrolled for microarray analysis, and the clinical and pathological characteristics are presented in Supplementary Table 1. Second, we enrolled consecutive 22 patients (9 women and 13 men, mean age 52.4 ± 10.3 years) with APA-KCNJ5 or APA-WT to endocrinological and pathological study. There were no patients with ATP2B3 or CACNA1D mutations. We excluded 2 APA samples with ATP1A1 mutation because of small number. The study flow chart is shown in Supplemental Figure 1. Written informed consent was obtained from all subjects. Our study was approved by the ethics committee of Hiroshima University. Small pieces of tissue sample in aldosterone producing adenoma were immediately preserved during surgery in RNAlater (Life Technology, Delhi, India) stored −80 °C until required, and whole tissues were fixed in formalin and embedded in paraffin for immunohistochemical analysis. APA was confirmed by detecting the expression of *CYP11B2* by immunohistochemistry and/or quantitative polymerase chain reaction assays as previously reported.^6^

### Genotyping

Genomic DNA from peripheral leucocytes and tissue samples were extracted and stored at −20 °C. Primer sequences specific for *KCNJ5*, *ATP1A1*, *ATP2B3*, and *CACNA1D* are summarized in Supplementary Table 2. PCR-based direct sequencing was performed as previously described.^23^ We defined APA without any known mutations as APA-wild type (APA-WT), and APA with a KCNJ5 mutation as APA-KCNJ5. Additionally, *CTNNB1* gene sequences in APA-WT were determined in the same manner.

### Endocrinological Evaluations

To alleviate the effects of adrenocorticotrophic hormone on aldosterone production, 1 mg of dexamethasone was orally administered to patients at 23:00, the night prior to the commencement of our study. A peripheral intravenous catheter was inserted before 7:30. Patients were rested in a supine position for at least 30 minutes before the start of testing. We administered 100 μg of GnRH (LH-RH, Mitsubishi Tanabe Pharma Corp., Osaka, Japan) at 08:00, and plasma aldosterone concentration (PAC) was measured 0, 15, 30 60, 90, and 120 minutes after the injection of GnRH. A GnRH loading test was performed for 9 APA-NM and 13 APA-KCNJ5 patients before they were operated upon. A positive response was defined as a greater than 50% increase in aldosterone following GnRH stimulation. An aldosterone response between 25% and 50% was evaluated as a partial response.

### RNA Extraction and Quantitative Polymerase Chain Reaction Assays

Total RNA was extracted using an RNeasy Mini kit (Qiagen, Hilden, Germany). For reverse transcription, 300 ng of total RNA was incubated with Takara PrimeScript RT Master Mix (Takara Bio Inc., Shiga, Japan) following the manufacturer's recommended protocol. The mRNA expression levels of *CYP11B2* and glyceraldehyde-3-phosphate dehydrogenase (*GAPDH*) were determined using a Taqman Gene Expression Assay kit (Applied Biosystems, Waltham, MA). The expression levels of *GNRHR* and *LHCGR* were assessed using SYBR green-based gene expression assays (Takara Bio SYBR Premix EX Taq; Takara Bio Inc.). The oligonucleotide primers we used to target *GNRHR* were 5′-TCA TCT AGC AGA CAG CTC TGG AC-3′ and 5′-ACC AAG AGC ACG GCT GAA GAC-3′. The primers for *LHCGR* were 5′-AGT CGT TAC AAA CTT ACA GTG CC-3′ and 5′-ATG GCA TGG TTA TAG TAC TGG C-3′.

### Microarray Analysis

Transcriptome data were generated by SurePrint G3 Human Gene Expression 8 × 60 K v2, which included approximately 50,000 unique probes (Agilent Technologies Inc., Santa Clara, CA). The gene set for GPCRs was extracted from the Gene Set Enrichment Analysis website (http://www.broadinstitute.org/gsea/index.jsp), and transcriptome data were analyzed by the R software package (University of Auckland).

### Statistical Analysis

Quantitative data are presented as the medians and interquartile ranges. Analyses were performed using SPSS for Windows (release 12.0; SPSS Inc., Chicago, IL), and *P* values <0.05 were considered to be significant. First, we performed Pearson correlation analysis for the relationship of GNRHR with PAC mediated by GnRH stimulation and LHCGR mRNA levels. Second, multiple regression analysis was conducted to investigate the association after adjustment for age, gender, serum K levels, and adenoma diameter. Third, unpaired comparisons in clinical and pathological characteristics were carried out by nonparametric Mann–Whitney *U* test. Finally, we tested the effect of KCNJ5 mutation on GNRHR mRNA expression by multiple regression analysis adjusted by age, gender, serum K level, and adenoma diameter.

## RESULTS

### Microarray Analysis of GPCR-Related Genes

**FIGURE 1:**
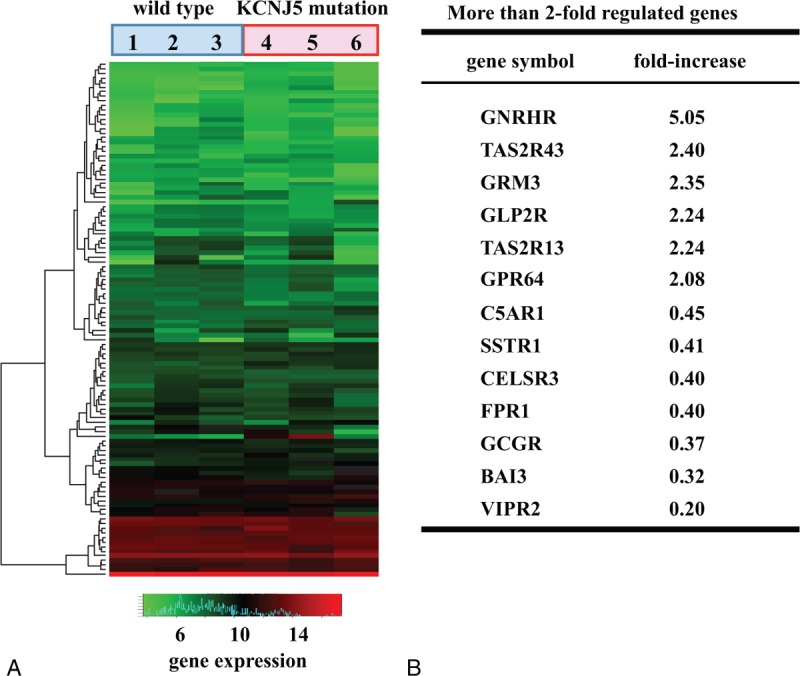
(A) Heat map generated from the microarray analysis of APA samples. GPCR-related gene sets were used in the analysis. (B) GPCR and GPCR-related genes that were differentially expressed by more than 2-fold in APA-WT samples compared with APA-KCNJ5 samples. APA = aldosterone-producing adenoma, APA-KCNJ5 = APA with the KCNJ5 mutation, APA-WT = APA without known mutations, GPCR = G protein-coupled receptor.

We conducted a microarray analysis of 192 GPCR and GPCR-related genes in 3 APA-WT and 3 APA-KCNJ5 samples (Figure 1A). We observed 13 genes with a more than 2-fold difference in the expression of genes in APA-WT samples than those in APA-KCNJ5 samples (Figure 1B). Of these differentially expressed genes, 6 were upregulated and 7 were downregulated in APA-WT.

### Clinical and Molecular Characteristics of APA-WT and APA-KCNJ5

**FIGURE 2:**
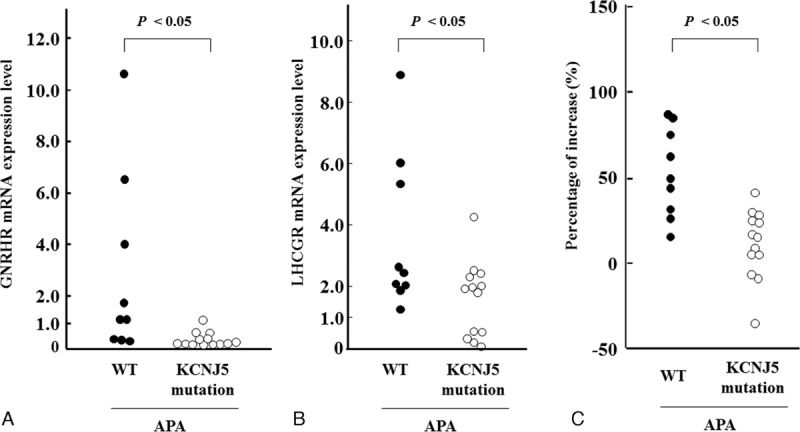
GNRHR (A) and LHCGR (B) mRNA expression levels between APA-WT (n = 9) and APA-KCNJ5 (n = 13) samples. (C) A comparison of aldosterone responses, as assessed by the GnRH loading test, between APA-WT and APA-KCNJ5 samples. APA-KCNJ5 = aldosterone-producing adenoma with the KCNJ5 mutation, APA-WT = APA without known mutations, GnRH = gonadotropin-releasing hormone, GNRHR = gonadotropin-releasing hormone receptor, LHCGR = luteinizing hormone/choriogonadotropin receptor, mRNA = messenger RNA.

**FIGURE 3:**
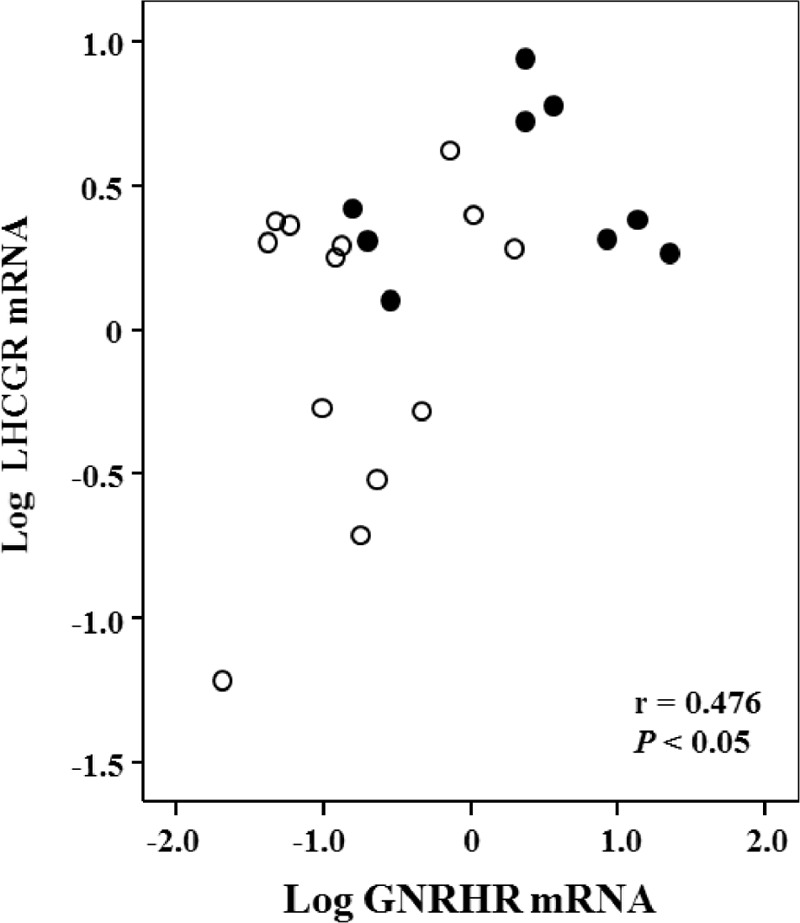
The relationship between GNRHR and LHCGR mRNA expression levels in APA samples. APA-WT and APA-KCNJ5 were indicated by closed circles and open circles, respectively. APA = aldosterone-producing adenoma, APA-KCNJ5 = aldosterone-producing adenoma with the KCNJ5 mutation, APA-WT = APA without known mutations, GNRHR = gonadotropin-releasing hormone receptor, LHCGR = luteinizing hormone/choriogonadotropin receptor, mRNA = messenger RNA.

The clinical characteristics of patients with APA-WT and APA-KCNJ5 are summarized in Table 1. GNRHR was the highest expression in APA-WT by our microarray analysis, and GNRHR was over-expressed in APA compared to normal adrenal tissue as previously reported.^17,19,24^ Therefore, we analyzed GNRHR mRNA expression levels between APA-WT and APA-KCNJ5 samples. APA-WT tissues exhibited higher levels of GNRHR expression than those in APA-KCNJ5 tissues (Figure 2A). In accordance with the previous reports which suggested that APA with higher GNRHR mRNA levels tended to have higher LHCGR mRNA levels,^17,19,24,25^ APA-WT had higher LHCGR levels compared with APA-KCNJ5 (Figure 2B). The expression levels of GNRHR correlated with those of LHCGR in 22 APA samples (Figure 3). Consistent with the relationship of mutation status with GNRHR and LHCGR mRNA levels (Figure 2A and B), patients with APA-WT had significantly higher increases in aldosterone levels than those with APA-KCNJ5 (Figure 2C). We observed an aldosterone response in 55.6% (5/9) of patients with APA-WT, while none of the APA-KCNJ5 patients exhibited an aldosterone response. A partial aldosterone response was seen in 22.2% (2/9) and 23.1% (3/13) of APA-WT and APA-KCNJ5 patients, respectively.

**TABLE 1:**
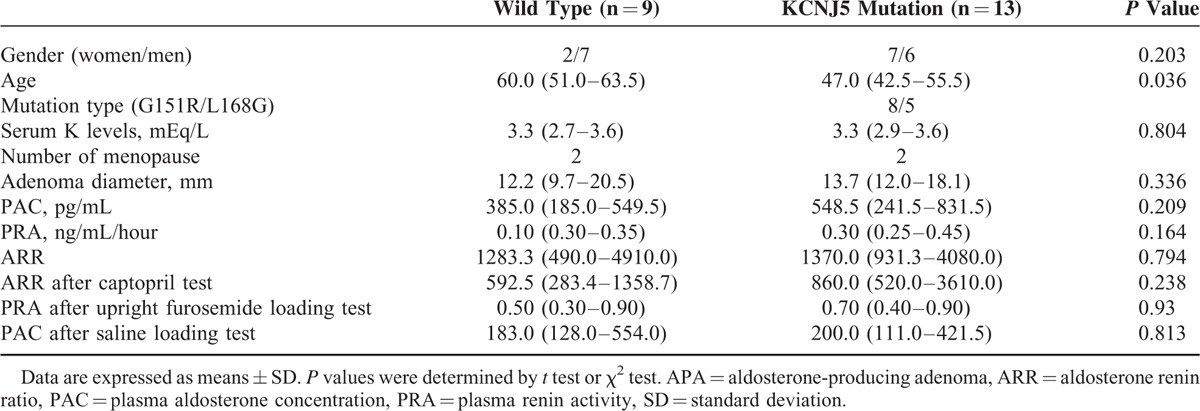
Clinical Characteristics Between APA With Wild Type and KCNJ5

There was no correlation with basal PAC and GNRHR or LHCGR mRNA levels. However, the percentage aldosterone increase due to by GnRH stimulation significantly correlated with GNRHR and LHCGR mRNA levels (Supplemental Figure 1A and B). Multiple regression analysis revealed a significant correlation between aldosterone increase and GNRHR mRNA levels that was independent of age, gender, serum potassium levels, and adenoma diameter (β = 0.242 and *P *< 0.01).

Recent study has demonstrated that GNRHR and LHCGR levels were abundantly expressed in APA tissues from the patients related with pregnancy, and the tissues had *CTNNB1* somatic mutation which code for β-catenin.^25^ However, our sequence analysis in APA-WT revealed no mutations in *CTNNB1* gene.

### Effects of KCNJ5 Mutation on GNRHR mRNA Expression

**FIGURE 4:**
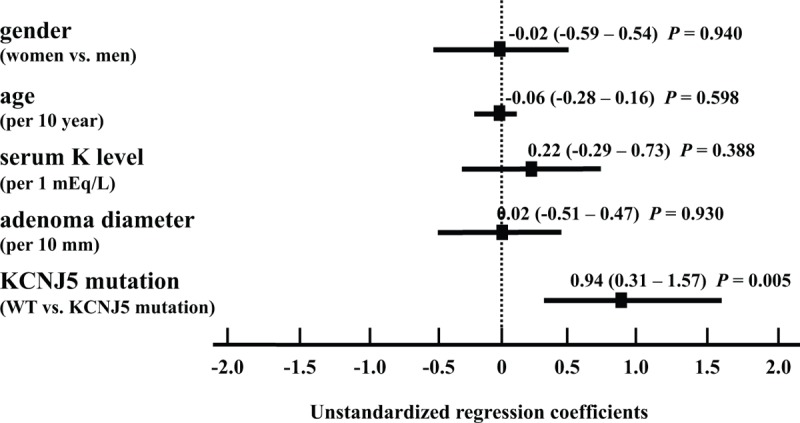
Unstandardized regression coefficients, 95% confident interval (CI), and *P*-values were determined using multivariate regression analysis of gender, age, serum potassium levels, and adenoma diameter.

**FIGURE 5:**
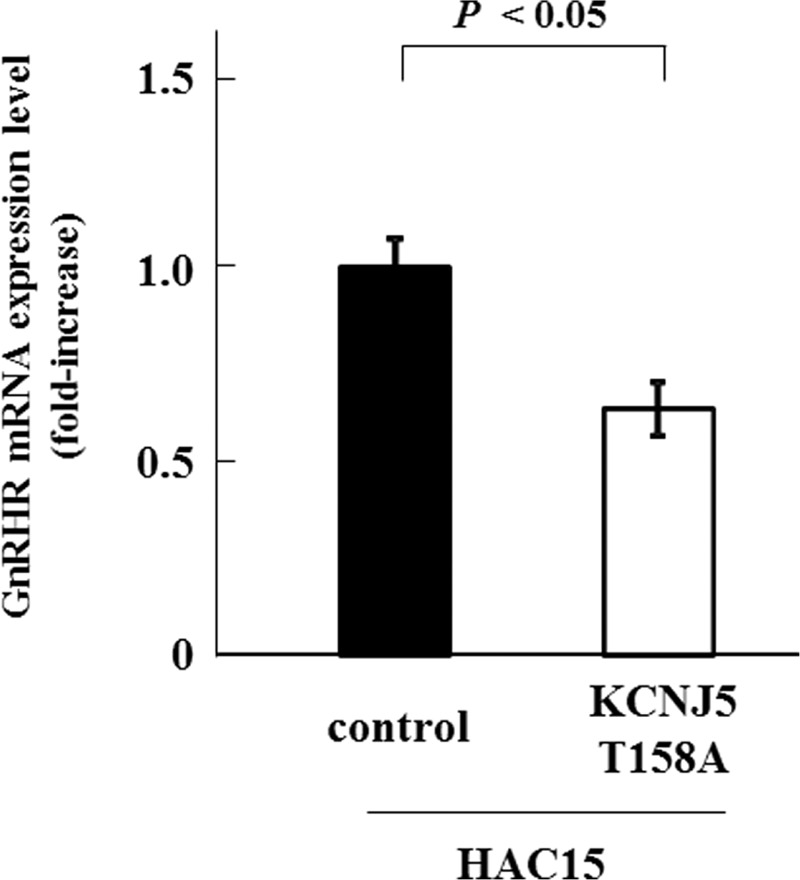
Effects of the KCNJ5-T158A mutation on GNRHR mRNA expression levels in HAC15 cells infected with control or KCNJ5-T158A lentiviruses. GNRHR = gonadotropin-releasing hormone receptor, mRNA = messenger RNA.

Multiple regression analysis revealed that the presence of the KCNJ5 mutation was negatively linked to GNRHR mRNA expression levels following adjustment for gender, age, serum potassium levels, and adenoma diameter (Figure 4). As the pathogenesis of APA-WT has not been clarified as yet, we investigated the effects of the KCNJ5 mutation on GNRHR expression levels in HAC15 cells. Expression levels of *CYP11B2* mRNAs in HAC15-T158A cells were elevated 17.7-fold in comparison with control cells,^12^ while GNRHR expression was reduced 0.64-fold compared with that in control cells (*P *< 0.05) (Figure 5). Taken together, our results indicate that the KCNJ5 mutation appears to result in a decrease in GNRHR expression levels.

## DISCUSSION

We demonstrated that GNRHR mRNAs were highly expressed among GPCR and GPCR-related genes in APA-WT samples using microarray analysis. GNRHR expression positively correlated with LHCGR expression in APA. Aldosterone increases by GnRH stimulation in APA-WT samples were higher than those in APA-KCNJ5 samples, correlating with the increased GNRHR and LHCGR mRNA levels. We also showed that the KCNJ5 mutation adversely affected GNRHR expression levels in clinical samples and in vitro.

In APA-WT, GNRHR expression in adenomas might participate in the regulation of aldosterone production. However, the pathogenesis and etiology of autonomous aldosterone production in these cases has not been fully elucidated. An increase in GNRHR expression levels was reportedly seen in 39.3% (11/28), 26.7% (4/15), and 27.3% (6/22) of APA samples.^17,19,24^ The overall prevalence and increase in GNRHR expression levels, we have reported in this study, are consistent with previous reports. In previous studies, the genetic background of the APAs were not reported. We postulate that in adenomas with no somatic mutations of the *KCNJ5* gene, that aberrant GNRHR expression regulates aldosterone production. However, the cause of this aberrant expression remains unknown. Our multivariate analysis and in vitro study results suggest that mutations in *KCNJ5* strongly influence GNRHR expression in a negative manner.

We also showed that patients with APA-WT, which have higher GNRHR and LHCGR expression levels, exhibited GnRH stimulation of aldosterone. Our results support those from a previous study, where APA patients with high GNRHR and LHCGR expression levels tended to show an aldosterone response to GnRH stimulation.^24^ Nakamura et al^26^ reported that GnRH stimulation potentiated *CYP11B2* expression and aldosterone production in H295R cells. Results from numerous studies have suggested that GPCRs, including GNRHR, stimulate adenylate cyclase and phospholipase C, which are important for aldosterone production.^27–29^ Aberrant GNRHR functions could regulate the production of aldosterone in APA, particularly in APA-WT cases. Therefore, the GNRHR-related pathway could be an important target with respect to the regulation of aldosterone production in a subset of APA.

We need to consider the relationship between GNRHR expression and tumor proliferation leading to APA. GnRH was found to activate MAPK signaling including ERK, JNK, p38, and BMK as well as adenylate cyclase and phospholipase C in pituitary derived cells.^30^ Of note, the activation of MAPK is one of the most important factors for cell proliferation and cell growth in adrenal cells.^31^ Moreover, GNRHR collaborates with other GPCRs for MAPK activation, and most of GPCRs have common mechanisms with GNRHR to activate MAPK via G proteins and Src family.^30,32^ In fact, metabotropic glutamate receptor 3 (GRM3), which is one of the highly expressed in our study, was also reported to stimulate MAPK,^33^ besides ectopic expression of taste receptor genes (TAS2R), glucagon like peptide 2 receptor (GLP2R), and GPR64 in tumors were also indicated to have association with tumor progression.^34–36^ Collectively, GNRHR as well as other GPCRs in APA-WT could participate the tumor progression, and we propose future basic studies with modulation of GPCRs expression to focus on cell proliferation in APA primary cell culture or zona-glomerulosa cells. Otherwise, this would assist in the development of novel molecular agents for the regulation of aldosterone production via tumor degradation.

Recent study suggested that Wnt signaling including β-catenin constitutive active mutation might arise a common adrenal-gonadal primordium and induce GNRHR expression.^25^ The study is consistent with our findings which showed low GNRHR levels in APA-KCNJ5. Although tissues from our study did not show the mutations in *CTNNB1*, our results do not deny the report because of the evidence that the activation of Wnt signaling is not associated with the presence of the mutation.^37,38^ Since the highly expressed GPCRs other than GNRHR are derived from ectoderm and endoderm, the embryonic primordium is different from its for GNRHR, mesoderm. The aberrant expression of GPCRs in APA-WT might be regulated after tumorigenesis, and thus there might be the intracellular or extracellular mechanisms for highly co-expression of GNRHR and other GPCRs.

The present study has several limitations. This study was cross-sectional, and then we could not establish a causal relationship among GNRHR expression, aldosterone production, and tumor progression. Second, our study subjects were limited only to 22 APA patients, because of a consequence of the cumbersome methods. Although a large-scale study is required to examine whether similar results would be obtained, our findings would be helpful to resolve the pathogenesis of APA. Third, our study did not have the patients related with pregnancy, and the number of patients showed menopause were limited. Renin–angiotensin–aldosterone system is affected by the pregnancy, menstrual cycle, menopause, or contraceptive therapy.^25,39–41^

We showed that GNRHR and LHCGR were highly expressed in some APA-WT samples, and that they positively correlated with GnRH-stimulated aldosterone production. Our findings regarding the aberrant receptor expression may be one of the candidates to elucidate the mechanisms of aldosterone production and/or the pathogenesis of APA-WT.

## Supplementary Material

Supplemental Digital Content

## Supplementary Material

Supplemental Digital Content
