# Supplementary material for: Gonadotropin-Releasing Hormone Stimulate Aldosterone Production in a Subset of Aldosterone-Producing Adenoma
Source: Medicine (Baltimore). 2016 May 20;95(20):e3659. doi: 10.1097/MD.0000000000003659 (PMC4902412; doi:10.1097/MD.0000000000003659)

**Supplemental methods**

**Diagnosis of primary aldosteronism (PA)**

The diagnosis of PA and the subtype diagnosis were performed according to the guidelines from the Japan Endocrine Society ^1^. Briefly, screening for PA was done by measuring plasma aldosterone concentration (PAC) and plasma renin activity (PRA) a ratio of aldosterone renin ratio >200 was applied. The definitive diagnosis were performed using 3 confirmation tests consisting of the captopril-challenge test, upright furosemide loading test, and saline loading test. To identify the localization of aldosterone hypersecretion, adrenal vein sampling technique under adrenocorticotrophic hormone stimulation was done and a PAC ≥14000pg/ml in adrenal vein and lateralization ratio ≥2.6 were applied for the subtype diagnosis.

**Clinical measurements**

The height, body weight, and waist girth of the subjects were measured using standard methods, and the BMI was calculated. Blood pressure was measured with a mercury sphygmomanometer after the subjects had rested in a sitting position for at least 10 minutes.

**Cell culture and Lentiviral infection**

The HAC15 human adrenocortical carcinoma cell line was provided by W. E. Rainey (University of Michigan). The HAC15 cells were cultured in Dulbecco’s modified Eagle’s medium (DMEM):F12 (1:1) supplemented with 10% Cosmic Calf serum (HyClone, Logan, UT) at 37C under an atmosphere of 5% CO2. pLX303-KCNJ5 T158A and control plasmid as pLX303 without KCNJ5 were prepared and lentiviral production and infection were performed as we previously reported ^2^.

**References**

1. Nishikawa T, Omura M, Satoh F, et al. Guidelines for the diagnosis and treatment of primary aldosteronism--the Japan Endocrine Society 2009. *Endocr J.* 2011;58:711-721.

2. Oki K, Plonczynski MW, Luis Lam M, Gomez-Sanchez EP, Gomez-Sanchez CE. Potassium channel mutant KCNJ5 T158A expression in HAC-15 cells increases aldosterone synthesis. *Endocrinology.* 2012;153:1774-1782.


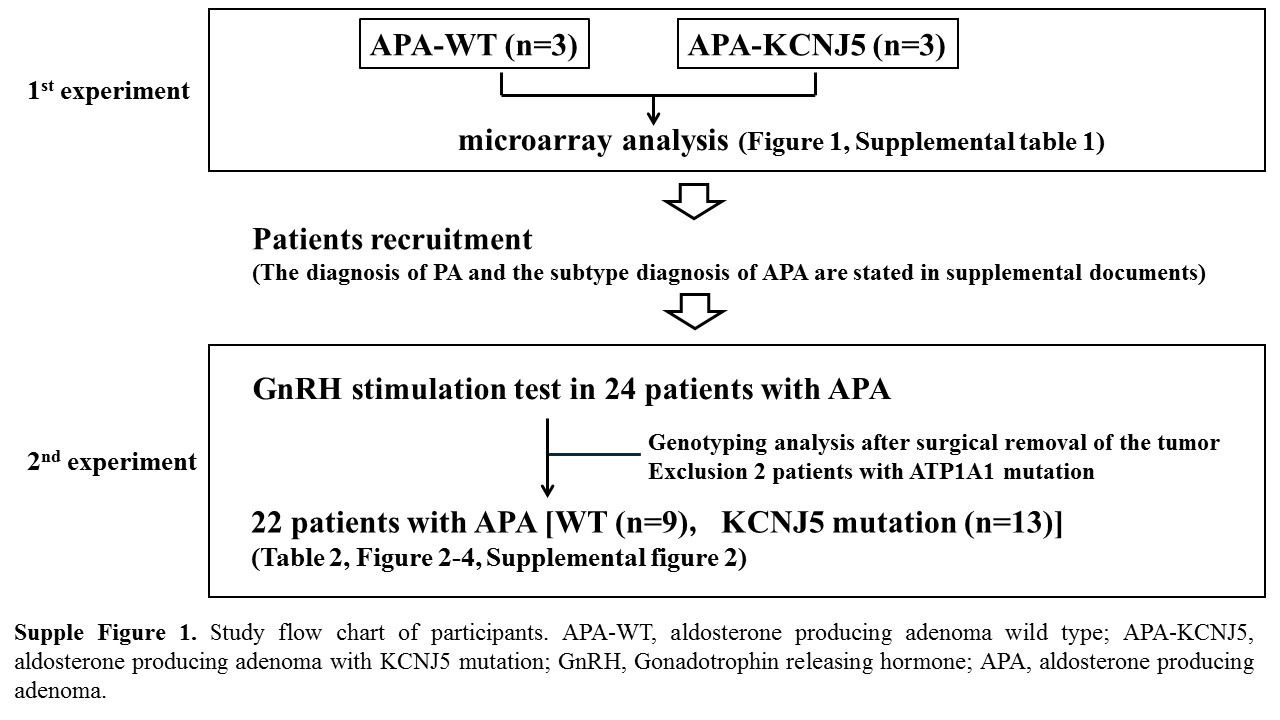


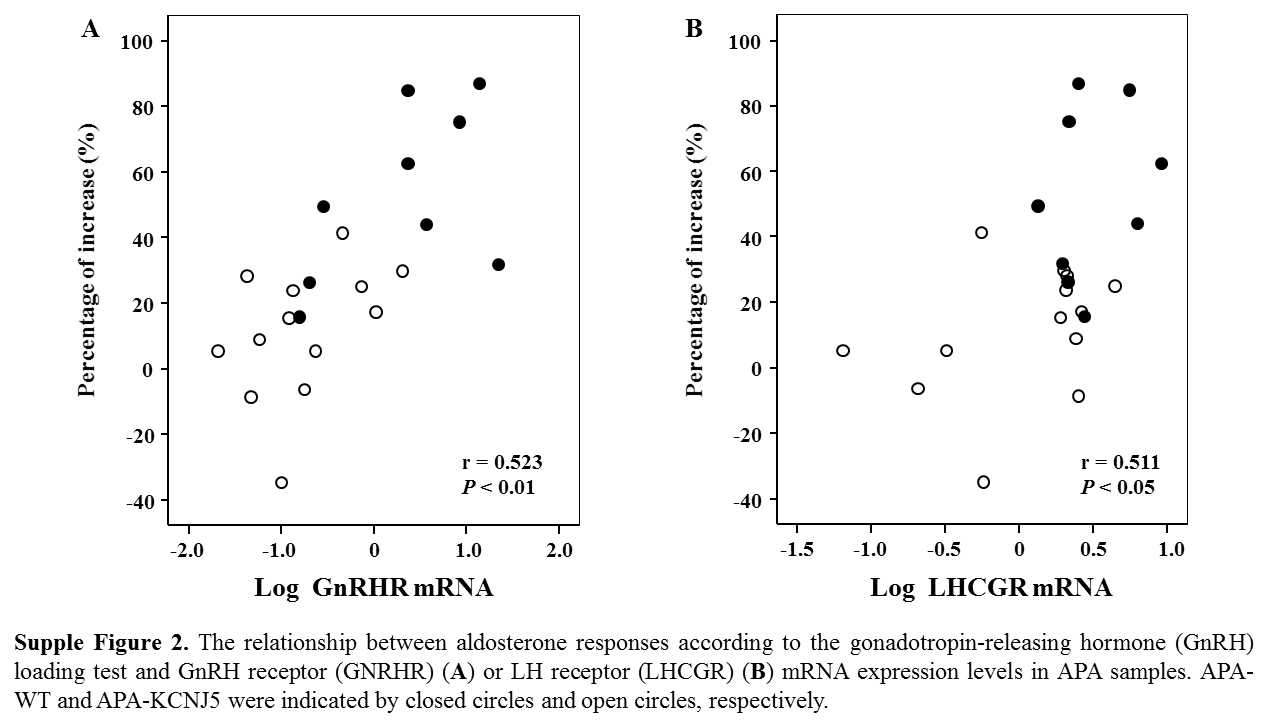

Supplement: Supplemental Digital Content [file medi-95-e3659-s001.doc]
