# Supplementary material for: Gonadotropin-Releasing Hormone Stimulate Aldosterone Production in a Subset of Aldosterone-Producing Adenoma
Source: Medicine (Baltimore). 2016 May 20;95(20):e3659. doi: 10.1097/MD.0000000000003659 (PMC4902412; doi:10.1097/MD.0000000000003659)
Supplement: Supplemental Digital Content [file medi-95-e3659-s002.pdf]

Supplemental Table 1. Clinical characteristics in the patients for microarray analysis

	gene mutation	gender (woman / man)	age	PAC (pg/ml)	PRA (ng/dl/hr)	ARR	Tumor location (Left / Right)	diameter (mm)	SBP (mmHg)	DBP (mmHg)	CYP11B2 (IH)	CYP11B2 (mRNA)
1	no mutation	man	46	358	0.6	597	Left	17.6	134	88	hetero	260.8
2	no mutation	woman	27	426	0.1	4260	Right	9.4	120	70	homo	449.3
3	no mutation	woman	32	277	0.4	692	Right	15.1	140	76	homo	317.1
4	KCNJ5 G151R	man	59	361	0.4	902	Left	16.4	168	70	hetero	306.3
5	KCNJ5 G151R	man	37	634	0.5	1268	Right	11.3	143	96	homo	466.1
6	KCNJ5 L168G	man	49	210	0.6	350	Right	12.7	115	67	hetero	95.6

PAC, plasma aldosterone concentration; PRA, plasma renin activity; ARR, aldosterone renin ratio; IH, immunohistochemistry; homo, positive homogeneously; hetero, positive heterogeneously. CYP11B2 mRNA expression levels were expressed as fold increase compared to those in 15 non-functioning cortical adenomas.

Supplemental table 2. Primers for PCR-based sequencing

Gene	Forward primer	Reverse primer
KCNJ5	5’-agtggcgcttcaacttgctcg-3’	5’-cgatgtgggagttgcggaggt-3’
ATP1A1	5’-accttggctctctagcttgggaca-3’	5’-tgcaggtgggcatcccact-3’
	5’-actggagagaagtccctttgccaaac-3’	5’-cacgtgatgtggctctcaagaagaact-3’
ATP2B3	5’-agcactccagaagaggccacacag-3’	5’-cgagggacctgagctgacagatca-3’
CACNA1D	5’-attcattggagtgccttcctggc-3’	5’-tcggggtggaaagggagactg-3’
	5’-agctgcaactggggctctgc-3’	5’-gcagagaagctgtcagggagcag-3’
	5’-accagcttcccgggatgca-3’	5’-gcctggcaggctgcaactg-3’
	5’-atggatcccacgctaactgtgca-3’	5’-cctcagctcagctctgcccag-3’
	5’-actgggcgtgcatgtggatg-3’	5’-gcatttggctgaaaaatcacaatgct-3’
CTNNB1	5’-aactgttaggtggttccctaagg-3’	5’-aacactcactatccacagttcagc-3’
